# Quasi-steady aerodynamic modeling and dynamic stability of mosquito-inspired flapping wing pico aerial vehicle

**DOI:** 10.3389/frobt.2024.1362206

**Published:** 2024-05-07

**Authors:** Balbir Singh, Kamarul Arifin Ahmad, Manikandan Murugaiah, Noorfaizal Yidris, Adi Azriff Basri, Raghuvir Pai

**Affiliations:** ^1^ Department of Aeronautical and Automobile Engineering, Manipal Institute of Technology, Manipal Academy of Higher Education, Manipal, Karnataka, India; ^2^ Department of Aerospace Engineering, Faculty of Engineering, Universiti Putra Malaysia, Serdang, Malaysia; ^3^ Aerospace Malaysia Research Centre, Faculty of Engineering, Universiti Putra Malaysia, Serdang, Malaysia; ^4^ Department of Mechanical and Industrial Engineering, Manipal Institute of Technology, Manipal Academy of Higher Education, Manipal, Karnataka, India

**Keywords:** flapping wing, pico aerial vehicle, trailing edge vortices, mosquitoes, quasi-steady modeling, flight control, kinematics, dynamic stability

## Abstract

Recent exploration in insect-inspired robotics has generated considerable interest. Among insects navigating at low Reynolds numbers, mosquitoes exhibit distinct flight characteristics, including higher wingbeat frequencies, reduced stroke amplitudes, and slender wings. This leads to unique aerodynamic traits such as trailing edge vortices *via* wake capture, diminished reliance on leading vortices, and rotational drag. This paper shows the energetic analysis of a mosquito-inspired flapping-wing Pico aerial vehicle during hovering, contributing insights to its future design and fabrication. The investigation relies on kinematic and quasi-steady aerodynamic modeling of a symmetric flapping-wing model with a wingspan of approximately 26 mm, considering translational, rotational, and wake capture force components. The control strategy adapts existing bird flapping wing approaches to accommodate insect wing kinematics and aerodynamic features. Flight controller design is grounded in understanding the impact of kinematics on wing forces. Additionally, a thorough analysis of the dynamic stability of the mosquito-inspired PAV model is conducted, revealing favorable controller response and maneuverability at a small scale. The modified model, incorporating rigid body dynamics and non-averaged aerodynamics, exhibits weak stability without a controller or sufficient power density. However, the controller effectively stabilizes the PAV model, addressing attitude and maneuverability. These preliminary findings offer valuable insights for the mechanical design, aerodynamics, and fabrication of RoboMos, an insect-inspired flapping wing pico aerial vehicle developed at UPM Malaysia.

## 1 Introduction

**FIGURE 1:**
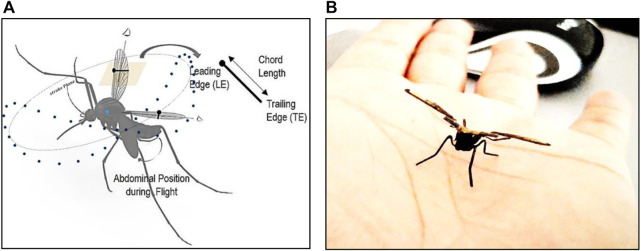
**(A)** Aerodynamic representation of mosquito during flight, mosquito-related flapping cycle phases, formation of leading and trailing edge vortices during phases of the wingbeat cycle of mosquito flight (Singh et al., 2021); **(B)** RoboMos, a mosquito-inspired FWPAV developed at UPM, Malaysia (Patented).

For decades, researchers have been fascinated by the flapping mechanism of insects and the development of insect-inspired flapping-wing micro aerial vehicles. Now, to develop these insect-based small flapping robots and controlstrategies, the morphology and flapping aerodynamic characteristics of the insects must be studied and modeled (Wang et al., 2020). Instead of focusing on an overall actuation system with wings, the emphasis should be on precisely designing the thorax-based flapping actuation mechanism. As shown in Figure 1A the thorax is an essential and influential section of an insect’s body that houses the wings and is packed with muscles and structures for wing-flapping operation and all power and control for excellent maneuverability (Madangopal et al., 2005). Mosquitoes have class aerodynamic characteristics, making them an exciting insect to study in biomimetics. They have slender wings, high flapping frequencies, low stroke amplitudes, and unique aerodynamic features such as trailing edge vortices for lift than traditional leading ones, rotational drag, delayed stall, and so on (Singh et al., 2021). Several studies have previously been conducted on real mosquitos and their exact computation models, both experimentally and through computational analysis, to better understand these mosquito mechanisms (Nakata et al., 2015; Bomphrey et al., 2017; Zhang and Huang, 2019; Liu et al., 2020). Researchers are also interested to see if there is any possibility of reconfigurable bio-inspired robots, even though it has been successfully tested on small drones (Harish Kumar et al., 2020). It is also critical to understand how the energy cost of small-sized insects varies with flight speed, as this is critical for performance (Zhu and Sun, 2020). The authors of this paper are aiming for a mosquito-inspired robotic insect, which is an electromechanical device propelled by a pair of symmetric flapping wings attached to a Nano-actuation system embedded in the thorax to achieve sustained autonomous flight and thus mimic an actual insect just like (Deng et al., 2006) and many others.

In this preliminary analysis, the quasi-steady aerodynamic modeling is given preference out of the available models (steady, quasi-steady, and unsteady). The complete details are added in Section 2. Steady models are not good at predicting loads on small insects, and unsteady models are not fully understood due to a lack of understanding of flow complexities at these low Reynolds numbers. As a result, the improved or modified quasi-steady models are preferred for preliminary analysis here, as they include all aerodynamic forces such as translational, rotational, added mass, and induced wake capture; however, it should be noted that these models must still be compared to data obtained from computational fluid dynamics and experiments (Xuan et al., 2020).

Reference (Nabawy and Crowther, 2014a) developed a generic quasi-steady aerodynamic model that can be applied to wings with arbitrary morphology and kinematics without experimental data. Later, the same authors in reference (Nabawy and Crowthe, 2015) developed a novel lifting-line theory that introduced the concept of the equivalent angle of attack and was capable of accurately estimating aerodynamic forces from any geometry and kinematic data. Apart from quasi-steady model analysis, it is noted that solving 3D Navier Stokes equations is vital to obtaining a clear picture of the aerodynamics behind these bio-creatures because these models alone fail to predict accurately the rotational lift term and the modelling of the wing-wake interaction (Pohly et al., 2018). As a result, quasi-steady alone can only be used for preliminary assessment and must be combined with computational analysis later to fully understand the unsteady aerodynamics associated with insect robot flight (Nakata et al., 2018; Ishihara, 2022).

This paper presents a comprehensive investigation into a mosquito-inspired flapping-wing Pico Aerial Vehicle (PAV) model, aiming to thoroughly understand its flight characteristics and stability before actual fabrication and testing (RoboMos, Figure 1B). Our primary objective was to assess whether mimicking mosquito wing kinematics and morphology, including wingbeat frequency and stroke amplitude derived from real mosquito studies (Bomphrey et al., 2017; Zhang and Huang, 2019; Liu et al., 2020), would translate to a stable flying robot at the pico scale.

To achieve this, we embarked on a multi-step analysis, we studied the wing motion, employing quasi-steady aerodynamic modeling for a rigid wing model. This analysis involved deriving the equations of motion and investigating the forces acting on the robot during flight. Following linearization, we determined the stability and control derivatives to understand the robot’s behavior in flight. This analysis revealed the inherent instability of the design based solely on mosquito-mimicking kinematics. Given the stability limitations, we explored the implementation of a quasi-steady-based non-linear controller to examine control behavior and performance. Our research drew inspiration from established works in the field. The model by Sane and Dickinson (2002) (Sane and Dickinson, 2002) provided a foundation for understanding the forces involved, while the controller design adapted code developed by Karasek et al. (2012); Karásek (2014) for hummingbird flight, with significant modifications for insect-scale flight dynamics. It is important to note that while these references focused on real-life flying creatures, our work centers on a miniaturized robotic model.

This combined approach, encompassing kinematic analysis, aerodynamic modeling, stability assessment, and controller design, serves as a crucial preliminary assessment for the RoboMos project. The findings from this paper will pave the way for improved design, fabrication, and control strategies for this bio-inspired flapping-wing PAV.

## 2 Mathematical modeling

Because of advances in Computational Fluid Dynamics (CFD), such as high-performance computing power, mathematical modeling of insect flapping flight has shifted from standard quasi-steady approximations to solving full-scale 3D Navier-Stokes equations for high-fidelity numerical studies. As mentioned in Section 1, CFD-based analysis is a promising tool. Still, its scope is limited in the case of insect flight due to the flow complexities associated with these creatures at such a low Reynolds number (Madangopal et al., 2005). It is highly unsteady. As a result, quasi-steady modeling aids in understanding the flow and calculating the forces and moments at the outset based on the given wing kinematics. The benefits of these models are that they can be easily incorporated into dynamic control models of these insects and can predict the energy and power requirements at the beginning, assisting in the design and development of insect robots (Madangopal et al., 2005).

**FIGURE 2:**
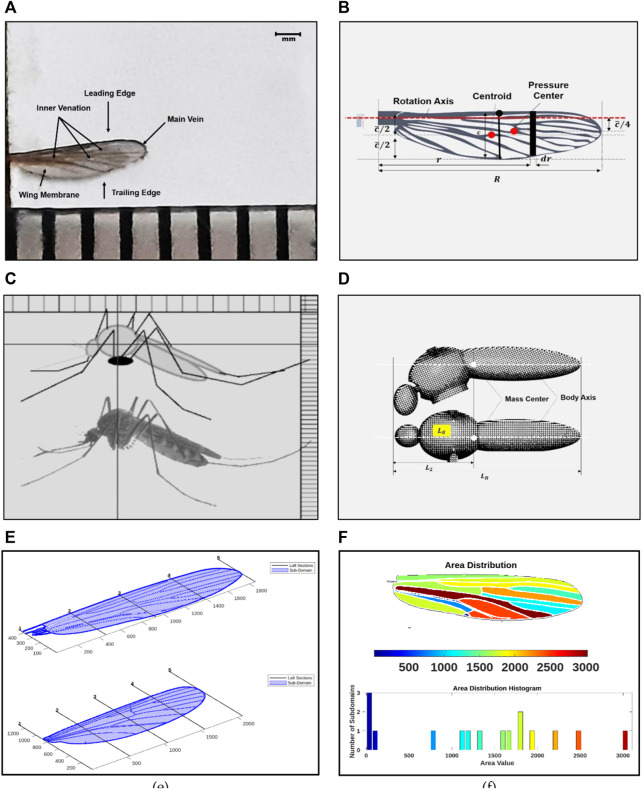
**(A)** Wing morphology including venation and corrugation of the real *Culex quinquefasciatus* wing; **(B)** Wing design modeled in design software and its geometric parameters **(C)** Body morphology of *Culex quinquefasciatus*; **(D)** Body design and geometric parameters of the PAV model **(E)** Morphology: Wing sample outline taking from real wing samples using computer vision **(F)** Wing area distribution and histogram given by the WingGram to understand the depth area.

Table 1 highlights the quasi-steady aerodynamic models developed from 1984 to 2022. It briefly mentions the type of forces included as part of the models for analysis. In this study, the wing morphology of an actual mosquito (*Culex quinquefasciatus*) found in peninsular Malaysia is used to design the PAV wing, as shown in Figures 2A, B and body in Figures 2C, D.

**TABLE 1:** Quasi-steady aerodynamic models were used to study the aerodynamics of flapping insect wings with mechanisms included (1984–2022). Reproduced with permission from (van Veen et al., 2022), Copyright 2022, The Cambridge University Press (UK).

[References]	Translational forces included	Rotational forces included	Added Mass			Wagner effect included	Wake capture included
			Included	Tangential forces	Variable virtual Mass		
Ellington (1984)	Yes	No	No^−^	-	-	No	No
DUDLEY and ELLINGTON (1990)	Yes	No	No	-	-	No	No
Sane and Dickinson (2002)	Yes	Yes	Yes	No	No	No	No^/^
(ANDERSEN et al., 2005) 2005	Yes	Yes	Yes	Yes	No	No	Yes
BERMAN and WANG (2007)	Yes	Yes	Yes	Yes	No	No	Yes
Liu (2009)	Yes	Yes	No	-	-	No	Yes
Nakata et al. (2015)	Yes	Yes	Yes	No	No	No	Yes
Dickinson and Muijres (2016)	Yes	Yes	No	-	-	No	No
Wang et al. (2016)	Yes	Yes	Yes	No	No	Yes^*^	No
Lee et al. (2016)	Yes	Yes	Yes	No	Yes	No	Yes
Van Veen et al. (2019)	Yes	Yes	No	-	-	No	No
Cai et al. (2021)	Yes	Yes	Yes	No	Yes	No	Yes
van Veen et al. (2022)	Yes	No^a^	Yes	Yes	Yes	Yes	No

^a^ This model focuses on wing stroke motion alone. - Added mass used for power requirement model and not for force model. * Applied only as a correction for accelerating wing and not for flapping./This model was based on pure kinematics without considering wake capture (stroke reversal) but tested on the wing with stroke reversal later.

The geometric positions, which include the position angles of all the veins, were marked with points. The wing corrugation is ignored here for the time being because it makes little difference to the aerodynamics of flight (Engels et al., 2020). WingGram developed by (Eshghi et al., 2022), a Matlab®-based open-source computer vision code, was used for initial wing modeling, as shown in Figures 2E, F. Following that, the mosquito wing and venation body were modeled in Autodesk^®^ Fusion 360 (Cheng and Sun, 2016; Lee et al., 2016). Figure 2B shows the wing geometric parameters; standard definitions are taken from the reference (Ellington, 1984). According to the definitions and after carefully scaling the mosquito-inspired PAV model based on the requirement, material availability, actuation, and transmission-related constraints, the final model has the following morphological parameters: ∼ m = 350 ± 50 mg (with legs); wing-length R = 13.01 mm; chord c = 3.01 mm, mean chord $\bar{c}$ = 2.61 mm, $r_{2}/R$ = 0.55; single wing area S = 67.92 mm^2^; body length $l_{b}$ = 14.01 mm. Dimensions of thorax for active actuation are 2.14 × 2.40 × 3.95 (mm). As in previous studies (Zhu et al., 2020), it is also assumed that the center of pressure (CP) is located near the rotational axis in the chord-wise direction. The product of the wing length R and the radius of the second moment of inertia $r_{2}$ determines the span-wise CP location.

### 2.1 Wing kinematic modeling and motion

As described in the previous section, the morphological characteristics like wing to body ratio, body angle of the real mosquito were used from the morphology study done. The kinematic characteristics of the hovering mosquito like its wingbeat frequency and stroke amplitude used for this PAV are based on previous experimental studies (Bomphrey et al., 2017; Zhang and Huang, 2019; Liu et al., 2020). Because the wing-body interaction is negligible, the model is assumed to be rigid and static, and the deformation of the wings in the span-wise variation of the pitching angle (Zhang and Huang, 2019) is not considered for this preliminary study. Deformation or wing flexibility is essential for lift generation and will be considered in the model’s complete 3D computational analysis in the future.

**FIGURE 3:**
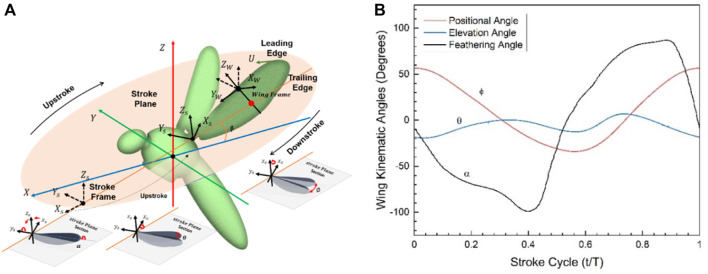
**(A)** PAV kinematic modeling and wing motion along the stroke plane; **(B)** General one full stroke cycle kinematics showing all the kinematic angles obtained by solving NS-equations; Positional ( ∅ ), Elevation ( θ ), and Feathering ( α ).

The full kinematic model of the robot model is shown in Figure 3A, which includes the global coordinate system ( X,Y,Z ) and the local Lagrangian type wing coordinate system ( $x_{s},y_{s},z_{s}$ ), with the origin at the wing base. The stroke plane and three Euler angles (positional angle, stroke deviation angle, and pitch angle) are defined in the kinematic model as follows (in the same way as done by (Liu and Sun, 2019)). The stroke plane angle ( β ) is the angle formed by the stroke plane and the X−Y plane (at hovering flight, X−Y plane is almost horizontal, β has a meager value). For simplicity, time during a cycle is always defined as a non-dimensional parameter, t/T , with t/T = 0 at the start of a downstroke and t/T = 1 at the end of the subsequent upstroke. The three flapping angles, the positional angle ∅ (related to stroke), the elevation angle θ (deviation angle), and the feathering angle α in terms of geometric angle of attack of wing in hovering are defined in Fourier series in Eqs 1–3 and behavior in Figure 3B:

$$\emptyset \left(t\right)=\sum_{n=0}^{3}\emptyset_{cn}\cos2knt+\emptyset_{sn}\sin2knt$$ (1)

$$\theta \left(t\right)=\sum_{n=0}^{3}\theta_{cn}\cos2knt+\theta_{sn}\sin2knt$$ (2)

$$\alpha \left(t\right)=\sum_{n=0}^{3}\alpha_{cn}\cos2knt+\alpha_{sn}\sin2knt$$ (3)

Table 2 shows the wing kinematic parameters defined for this hovering PAV model. Previous literature reveals that mosquito wings are located above the mass center, an essential point for this analysis and for developing this mosquito-inspired PAV model. The position of the legs (spread or not spread) does not affect the center of mass but does affect the moment of inertia (Liu and Sun, 2019).

**TABLE 2:** Wing kinematic parameters as defined for this model in the hovering state.

ϕ	f	S	AR	β	J ^a^	k	Re
44°	777 Hz	67.92 mm^2^	9.97	3°–8°	0.01	0.410	2,600

^a^ Lowest value of advanced ratio $\left(J=V/2\phi fR\right)$ indicates hovering. St - not defined (hovering) and $Re=\frac{4\phi fR^{2}}{\gamma \left(AR\right)}$

**FIGURE 4:**
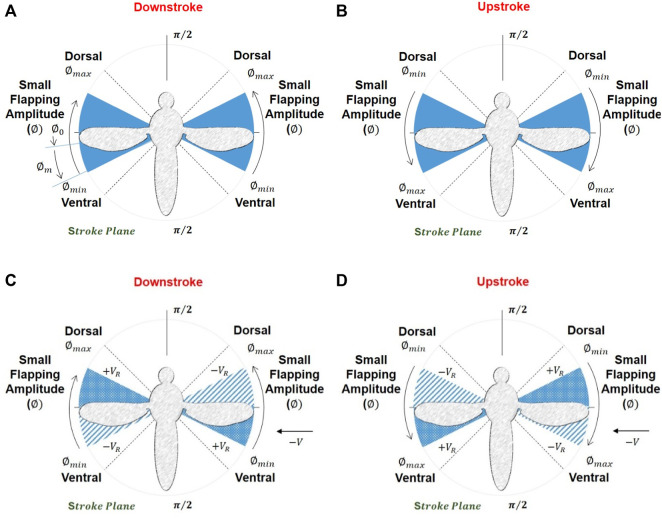
**(A, B)** Maximum and minimum stroke amplitude and the small amplitude angle the PAV possesses **(C, D)** variation in relative velocity during the downstroke and upstroke (stroke cycle) (top view). (+) sign indicates an increase and (−) for a decrease in relative velocity during the stroke cycle.

Figures 4A, B show the minimum and maximum range of stroke amplitude for the mosquito model, which is very small, and Figures 4C, D show the variation of relative velocity $V_{R}$ during the downstroke and upstroke (stroke cycle from the top view). Generally, hovering is accomplished at a symmetric flapping speed where the total average lift force from two wings equals the body weight and passes through the center of mass without inducing any torque (Zhang et al., 2020). A complete understanding of aerodynamic modeling is crucial for the control simulators used (Karásek and Preumont, 2012; Karásek, 2014). Insect wing motion is complex, not a simple sinusoidal or harmonic motion along its four phases. Still, it is assumed simple harmonic here for simplicity based on the following equations: (4), (5) and (6). At the extreme flap, the positional angle ∅ goes maxima ( $\emptyset_{\max}$ ) and minima ( $\emptyset_{\min}$ ). Thus, stroke amplitude ϕ, therefore, is the difference between these maxima and minima, and the mean (positional) stroke angle $\bar{\emptyset }$ is their average.

$$\emptyset =\emptyset_{0}+\emptyset_{m}\cos2\pi ft$$ (4)

$$\emptyset =\bar{\emptyset }+\frac{\phi }{2}\cos2\pi ft$$ (5)

$$\alpha =\alpha_{0}+\left(\frac{\pi }{2}-\alpha_{m}\right)\sin2\pi ft-\cos\left(2\pi ft-\varphi \right)$$ (6)

### 2.2 Quasi-steady aerodynamic approximation

Due to the complexity and unsteady flow characteristics of insect wings, aerodynamic modeling methods continue to face significant difficulties and challenges. The authors of the reference (Xuan et al., 2020) provided a beautiful explanation of these models, stating their classification into three types: steady-state, quasi-steady, and unsteady. Steady-state models are simple and inappropriate for predicting load in small insects during flight. Quasi-steady models are good predictive models because they account for translational, rotational, and added mass forces and circulatory and non-circulatory components in all aerodynamics associated with these bio-creatures. Their output, however, differs slightly from that of computational fluid dynamics or experiments. Applying full-scale unsteady models to such complex flows necessitates additional research.

This paper employs a fundamental analysis of a mosquito-inspired PAV model, utilizing a simple quasi-steady approach rooted in the semi-empirical model developed by (Sane and Dickinson, 2002). In this analysis, the wing is considered to be rigid and flat, with the model grounded in blade element theory. Forces such as those arising from added mass and wake capture, as represented in Eqs 7, 8, are disregarded due to their minimal impact on overall force. Instead, the focus lies on pressure distribution-derived forces and their influence on movement, encompassing translation and rotation, as discussed by Karásek and Preumont (2012).

It is important to remember that the availability of appropriate flapping force coefficients from experimental data is critical to the success of a quasi-steady aerodynamic model (Nabawy and Crowther, 2014b). According to reference (Xuan et al., 2020), the total instantaneous force, therefore, can be expressed as:

$$F_{inst}=F_{trans}+F_{rot}+F_{wc}+F_{am}$$ (7)

$$F_{am}=\rho \frac{\pi }{4}R^{2}{\bar{c}}^{2}\left(\ddot{\emptyset }\sin\alpha +\dot{\emptyset }\dot{\alpha }\cos\alpha \right)\int_{0}^{1}\hat{r}{\hat{c}}^{2}\left(\hat{r}\right)dr-\ddot{\alpha }\rho \frac{\pi }{16}{\bar{c}}^{3}R\int_{0}^{1}{\hat{c}}^{2}\left(\hat{r}\right)dr$$ (8)

According to reference (Deng et al., 2006; Karásek and Preumont, 2012), the total force is defined as components of the force in normal and tangential directions of the wing as in Eqs 9, 10:

$$F_{trans-T}=\frac{1}{2}\rho S{U_{CP}}^{2}C_{T}\left(\alpha \right)$$ (9)

$$F_{trans-N}=\frac{1}{2}\rho S{U_{CP}}^{2}C_{N}\left(\alpha \right)+\pi \left(\frac{3}{4}-{\hat{x}}_{0}\right)\rho \dot{\alpha \frac{U_{CP}}{{\hat{r}}_{2}}{\bar{c}}^{2}R\int_{0}^{1}\hat{r}{\hat{c}}^{2}\left(\hat{r}\right)d\hat{r}}$$ (10)

Where ρ is the air density and $C_{N}\left(\alpha \right)$ and $C_{T}\left(\alpha \right)$ are the force coefficients given as a function of angle of attack α by expressions in Eq. 11 (Karásek and Preumont, 2012; Karásek, 2014)

$$\begin{array}{c} C_{T}\left(\alpha \right)=\left\{\begin{array}{l} 0.4\cos^{2}\left(2\alpha \right),0\leq \left|\alpha \right|<\frac{\pi }{4}\\ 0,\frac{\pi }{4}\leq \left|\alpha \right|<\frac{3\pi }{4}\\ -0.4\cos^{2}\left(2\alpha \right),\frac{3\pi }{4}\leq \left|\alpha \right|<\pi \end{array}\right.\\ C_{N}\left(\alpha \right)=3.4\sin\left(\alpha \right) \end{array}$$ (11)

### 2.3 Model dynamics

The PAV model dynamics are represented using a standard averaged model, and linearization is accomplished using the small perturbation method. The aerodynamic derivatives have also been calculated by simply solving the Navier-Stokes equations. There are also analytical ways to calculate the derivatives. Finally, the model’s stability properties are investigated at the hovering state at a very low Reynolds number.

Because the quasi-steady model is non-linear and the insect model is a time-variant dynamic system, the oscillating mass distribution and periodic aerodynamic and inertial forces associated with flapping wings can couple with the insect model’s natural modes of motion (Zhu et al., 2020). This coupled phenomenon can only be ignored or is unlikely to occur if the flapping frequency is extremely high, as with mosquitoes (Zhu et al., 2020). In this analysis, the forces are replaced with average flapping cycle forces, representing the model as a cycle-averaged model (Karásek and Preumont, 2012; Zhu et al., 2020). Therefore, the insect model is rigid in this analysis, and forces and moments are time-averaged over the flapping period.

**FIGURE 5:**
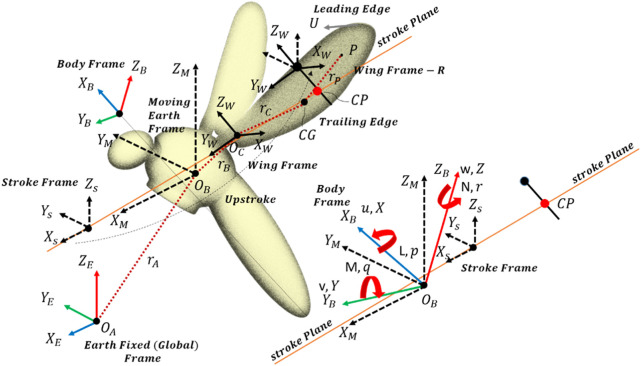
Dynamical modeling of the mosquito-inspired RoboMos PAV model.

The equations of motion (conservation of linear momentum equations CLMEs, conservation of angular momentum equations CAMEs, and kinematic equations are considered similar to that of an aircraft or helicopter and can be found in any standard book on flight dynamics and control like (Napolitano, 2011; Stevens et al., 2016). These equations in their non-linear form are related to the dynamic modeling of the body and wing of the mosquito-inspired PAV as shown in Figure 5 (Four frames of reference: earth-fixed ( $X_{E}$ , $Y_{E}$ , $Z_{E}$ ), earth-moving ( $X_{M}$ , $Y_{M}$ , $Z_{M}$ ), body-axis ( $X_{B}$ , $Y_{B}$ , $Z_{B}$ ), and wing-axis ( $X_{W}$ , $Y_{W}$ , $Z_{W}$ ); velocities u , v and w ; Linear momentums L , M and N ; angular rates p , q and r ). These non-linear equations must be linearized using a small perturbation method, then factorized with stability derivatives and non-dimensionalized to obtain longitudinal and lateral system matrices, respectively (Zhang and Sun, 2010; Xu and Sun, 2013; Zhu et al., 2020).

### 2.4 Simplifying equations of motion and dynamics

The equations of motion given below for this flapping Pico aerial vehicle have been originally developed and presented by reference (Gebert et al., 2002) and error corrected by reference (Sun et al., 2007). The authors have used these general equations from the above references for this PAV as per their PAV dynamic model shown in Figure 5. The velocity for each frame is given in Eq. 12:

$$V_{E}=\left[\begin{array}{c} V_{X_{E}}\\ V_{Y_{E}}\\ V_{Z_{E}} \end{array}\right],V_{B}=\left[\begin{array}{c} V_{X_{B}}\\ V_{Y_{B}}\\ V_{Z_{B}} \end{array}\right]\text{ and }V_{W}=\left[\begin{array}{c} V_{X_{W}}\\ V_{Y_{W}}\\ V_{Z_{W}} \end{array}\right]$$ (12)

Where $V_{X_{B}}$ are the components of velocity V in each frame. In terms of direction cosine matrices E , these equations can be written as in Eq. 13:

$$V_{B\to E}=E_{B\to E}V_{B},V_{W\to E}=E_{W\to E}V_{W}\text{ and }V_{W\to B}=E_{W\to B}V_{W}$$ (13)

B→E , W→E, and W→B represent the coordinate frame transformations. Two sets of equations define the insect PAV motion: equations of motion and fluid dynamic equations. Both sets of equations and their derivation are provided in references (Gebert et al., 2002; Sun et al., 2007) and will not be repeated here. Let $F_{A_{T}}$ and $M_{A_{T}}$ be the total aerodynamic force and moment about the center of gravity (CG) of the PAV model. $m_{T}$ be the total PAV mass, $m_{W}$ the mass of the wing, $I_{B}$ be the Inertia tensor matrix for the body, $I_{W}$ be the Inertia tensor matrix for the wing, g be the acceleration due to gravity and t is the time. Referring to Figure 5, $v_{cg}$ is the velocity of the center of gravity, $\omega_{B}$ is the angular velocity of body rotation, $\omega_{W}$ is the angular velocity of wing rotation, $r_{A}$ is the position vector from CG to the wing root, $r_{C}$ is the position vector from wing root to wing CG, and $r_{P}$ is the position vector from wing CG to any generic point P on the wing. Both translational and rotational equations can be written as Eqs 14, 15:

FATB+mB+∑i=1NmW,igB=mB+∑i=1NmW,idvcgBdt+ωBB×vcgB+∑i=1NmWdωBBdt×rBB+ωBB×ωBB×rBBi+∑i=1NmWEW→BdωWWdt×rCW+ωWW×ωWW×rCWi (14)

MATB+∑i=1NmWrBB+rCB×gBi=ωBB×IBBωBB+ddtIBBωBB+∑i=1NmWrBB+rCBvcgB+ωBB×rBB+mWrBB×ωWB×rCB+EW→BIWWωWWi+∑i=1NωBB×EW→BIWWωWW+mWωBB×rBB×ωWB×rCB+mWωBB×rBB+rCB×vcgB+ωBB×rBB+mWvcgB×ωBB×rBB+ωWB×rCBi (15)

Assuming a rigid body, Eqs 14,15 can be simplified further. Let $\omega_{W0}$ be the angular velocity of the wing relative to the body determined by the flapping motion of the wing so that ωWW=ωW0W+EB→WωBB and ωWB=EW→BωWW=ωW0B+ωBB . Using these two formulations in Eqs 12, 13 with further simplification (two symmetric wings N= 2), we have simplified Eqs 16, 17:

FATB+mBgB=mBdvcgBdt+ωBB×vcgB+A1+B1 (16)

MATB=ωBB×IBBωBB+IBBdωBBdt+A2+B2 (17)

Where ${\;A}_{1},B_{1}$ , $A_{2},B_{2}$ can be defined using Eqs 18–21.

A1=mW∑i=12−gB+dvcgBdt+ωBB×vcgB+dωBBdt×rBB+rCB+ωBB×ωBB×rBB+rCBi (18)

B1=mW∑i=12EW→BE˙B→WωBB+EW→BdωW0Wdt×rCB+ωBB+ωW0B×ωW0B×rCB+ωW0B×ωBB×rCBi (19)

A2=∑i=12mWrBB+rCB×−gB+dvcgBdt+dωBBdt×rBB+mWrBB×dωBBdt×rCB+mWvcgB×ωBB×rBB+rCB+mWωBB×rBB+rCB×vcgB+ωBB×rBB+rBB×ωBB×rBB+EW→BIWEB→WdωBBdt+ωBB×EW→BIWWEB→WωBBWi (20)

B2=∑i=12mWrBB×dωW0Bdt×rBB+ωBB+ωW0B×E˙W→BrCW+E˙W→BIWWωW0W+EB→WωBB+EW→BIWWdωW0Wdt+ωBB×EW→BIWWωW0W+EW→BIWWE˙B→WωBB+mWωBB×rBB×ωW0B×rCB+mWE˙W→BrCW×vcgB+ωBB×rBB+mWvcgB×ωW0B×rCBi (21)

Usually, for this PAV model, the $m_{W}$ remains much smaller than $m_{B}$ , comparing the body weight mBgB and body inertial force dvcgBdt+ωBB×vcgB , Eqs 16, 17 can be simplified further to obtain Eqs 22, 23 as:

FATB+mBgB=mBdvcgBdt+ωBB×vcgB+B1 (22)

MATB=ωBB×IBBωBB+IBBdωBBdt+B2 (23)

Using references (Gebert et al., 2002; Sun et al., 2007), given the assumption of a rigid body, the wing’s flapping frequency is notably high, resulting in a flapping motion time scale significantly shorter than that of the body’s motion. In the context of fluid dynamics, we can employ averaged forces, encompassing both aerodynamic and inertial forces, operating over the time scale of the body. By averaging Eqs 22, 23 over the flapping period to filter out rapid motions and capture the slower time scale, we derive Eqs 24, 25 as presented below:

FATB+mBgB=mBdv¯cgBdt+ω¯BB×v¯cgB+ω^BB×v^cgB+B¯1 (24)

MATB=ω¯BB×IBBω¯BB+ω^BB×IBBω^BB+IBBdω¯BBdt+B¯2 (25)

Where $\hat{v}$ and $\hat{\omega }$ represent fast body flapping due to cyclic variations of forces and moments at a particular flapping frequency, as the flapping motion is assumed to be rapid, oscillations are anticipated to be minimal. Consequently, we can disregard specific terms, leading to the approximation that ${\bar{B}}_{1}$ and ${\bar{B}}_{2}$ are nearly zero. This is because these average inertial forces and moments linked to flapping entail acceleration and deceleration phases that offset each other within one cycle. In the most straightforward rendition, Eqs 26, 27 become the most basic forms of translational and rotational equations for this PAV during slow-time-scale motion, mirroring the principles found in the equations governing a rigid aircraft (Gebert et al., 2002; Sun et al., 2007).

FATB+mBgB=mBdv¯cgBdt+ω¯BB×v¯cgB (26)

MATB=ω¯BB×IBBω¯BB+IBBdω¯BBdt (27)

### 2.5 Linearization, stability matrices and derivatives

**FIGURE 6:**
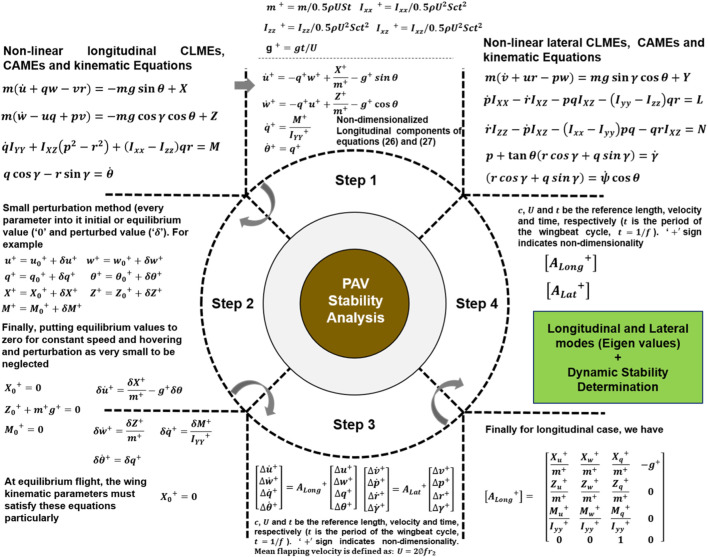
The general process of obtaining the longitudinal and lateral directional eigenvalues from system matrices is to get the system modes and determine the dynamic stability characteristics.

Eqs 26, 27 can now be utilized to determine the stability of this PAV model. For stability analysis, these CLMEs, CAMEs, and KEs must first be linearized using a small perturbation method for longitudinal and lateral motion. Figure 6 depicts the entire process.

There are five essential points to note about the process displayed in Figure 6 and the approach used in this study. First, it is impossible to obtain system matrices in Eqs 28, 29 without total mass, moment of inertia, and stability derivatives. Second, wing kinematics are required to calculate stability derivatives, and morphological parameters (such as the position of the center of mass, the distribution of total mass, and the distance between the wing base and the center of mass) are required to calculate total mass and moments of inertia.

For this mosquito-inspired pico aerial vehicle model analysis, wing kinematics and morphological parameters are derived, taking reference from (Bomphrey et al., 2017; Liu and Sun, 2019), respectively. Third, the body’s position concerning the body frame of reference changes when the flight speed changes. As a result, the moment and product of inertia change as well. However, it is neglected here because of hovering conditions.

$${A_{Long}}^{+}=\left[\begin{array}{cccc} \frac{{X_{u}}^{+}}{m^{+}} & \frac{{X_{w}}^{+}}{m^{+}} & \frac{{X_{q}}^{+}}{m^{+}} & -g^{+}\\ \frac{{Z_{u}}^{+}}{m^{+}} & \frac{{Z_{w}}^{+}}{m^{+}} & \frac{{Z_{q}}^{+}}{m^{+}} & 0\\ \frac{{M_{u}}^{+}}{{I_{yy}}^{+}} & \frac{{M_{w}}^{+}}{{I_{yy}}^{+}} & \frac{{M_{q}}^{+}}{{I_{yy}}^{+}} & 0\\ 0 & 0 & 1 & 0 \end{array}\right]$$ (28)

$${A_{Lat}}^{+}=\left[\begin{array}{cccc} \frac{{Y_{v}}^{+}}{m^{+}} & \frac{{Y_{p}}^{+}}{m^{+}} & \frac{{Y_{r}}^{+}}{m^{+}} & g^{+}\\ \frac{{I_{zz}}^{+}{L_{v}}^{+}+{I_{xz}}^{+}{N_{v}}^{+}}{{I_{xx}}^{+}{I_{zz}}^{+}-{{I_{xz}}^{+}}^{2}} & \frac{{I_{zz}}^{+}{L_{p}}^{+}+{I_{xz}}^{+}{N_{p}}^{+}}{{I_{xx}}^{+}{I_{zz}}^{+}-{{I_{xz}}^{+}}^{2}} & \frac{{I_{zz}}^{+}{L_{r}}^{+}+{I_{xz}}^{+}{N_{r}}^{+}}{{I_{xx}}^{+}{I_{zz}}^{+}-{{I_{xz}}^{+}}^{2}} & 0\\ \frac{{I_{xz}}^{+}{L_{v}}^{+}+{I_{xx}}^{+}{N_{v}}^{+}}{{I_{xx}}^{+}{I_{zz}}^{+}-{{I_{xz}}^{+}}^{2}} & \frac{{I_{xz}}^{+}{L_{p}}^{+}+{I_{xx}}^{+}{N_{p}}^{+}}{{I_{xx}}^{+}{I_{zz}}^{+}-{{I_{xz}}^{+}}^{2}} & \frac{{I_{xz}}^{+}{L_{r}}^{+}+{I_{xx}}^{+}{N_{r}}^{+}}{{I_{xx}}^{+}{I_{zz}}^{+}-{{I_{xz}}^{+}}^{2}} & 0\\ 0 & 1 & 0 & 0 \end{array}\right]$$ (29)

Fourth, to compute the stability and control derivatives, a common approach has been adopted for insects in many studies before, like in (Sun and Tang, 2002; Mou and Sun, 2012), references (Zhang and Sun, 2010; Karásek and Preumont, 2012; Liu and Sun, 2019; Zhu et al., 2020) and for Cranefly in reference (Xu et al., 2021), the description of which is not repeated here again. A simple immersed boundary method-based OpenFoam solver is used here for the relative motion of the wings and for calculating derivatives, followed by high-fidelity CFD analysis based on volume penalization (Engels et al., 2019).

**FIGURE 7:**
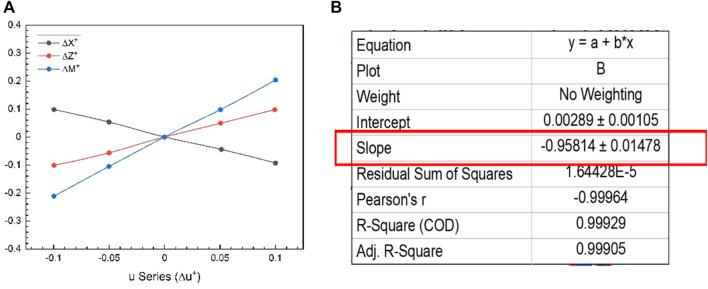
**(A)** Longitudinal stability derivatives from u -series forces and moments data (values of ${{\;X}_{u}}^{+}$ , ${Z_{u}}^{+}$ and ${M_{u}}^{+}$ are obtained from each curve); **(B)** slope of the $∆X^{+}$ by linear fitting in u -series to get the value of ${X_{u}}^{+}$ .

In short, the methodology is to choose one parameter that varies around hover and keep the others in equilibrium. The cycle average forces and moments around each value are then calculated and plotted as a function of the parameter of interest. The linearity in the graphical results also justifies the linearization process used here, as shown in Figure 7A. The slopes of tangents in the origin to the curves obtained are the stability derivatives, as shown in Figure 7B. The control derivatives are also brought in the same way. Fifth, as stated at the outset of this section and from reference (Karásek and Preumont, 2012), it would be necessary to design a robot with symmetrical mass distribution around all three-body axes to avoid the coupling between motions in longitudinal and lateral cases. Stability derivatives can also be obtained using analytical methods, for example, a local averaging method (Orlowski and Girard, 2011). Table 3 shows the values obtained for each non-dimensional longitudinal and lateral dynamic stability derivative from this dynamics approach. Forces and moments of both wings and body are essential for getting derivatives in hovering flight, but body contribution can be neglected because of negligible velocity and minimal interaction between body and wings (Aono et al., 2008).

**TABLE 3:** Non-dimensional longitudinal and lateral stability derivatives for our model.

Insect/Model	${X_{u}}^{+}$	${Z_{u}}^{+}$	${M_{u}}^{+}$	${X_{w}}^{+}$	${Z_{w}}^{+}$	${M_{w}}^{+}$	${X_{q}}^{+}$	${Z_{q}}^{+}$	${M_{q}}^{+}$
RoboMos (M)	−0.958	1.011	2.066	0.410	−2.620	−0.406	−0.649	0.186	−0.428

Insect/Model	${Y_{v}}^{+}$	${L_{v}}^{+}$	${N_{v}}^{+}$	${Y_{p}}^{+}$	${L_{p}}^{+}$	${N_{p}}^{+}$	${Y_{r}}^{+}$	${L_{r}}^{+}$	${N_{r}}^{+}$
RoboMos (M)	−1.416	1.262	0.757	−0.168	−3.763	−0.709	−0.480	0.320	−3.758

Aerodynamic forces and moments at various flight speeds can be calculated using kinematics at equilibrium flight. In general, at hover or even at very low velocity, these non-dimensional forces and moments ${{\;F}_{Z}}^{+}$ , ${F_{x}}^{+}$ and ${M_{Z}}^{+}$ have a non-zero contribution from the wings and a near-zero contribution from the body.

## 3 Control implementation and strategy

**FIGURE 8:**
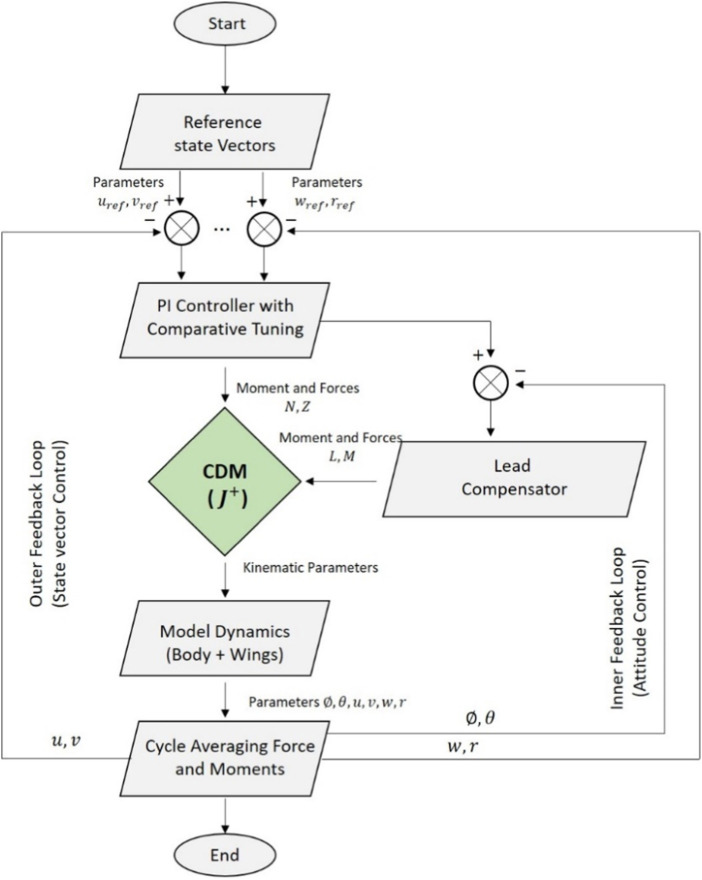
Controller flowchart for the simulator (Karásek and Preumont, 2012) with all the subsystems and control loops for velocity and attitude control.

For this analysis, we followed the control strategy developed by (Karásek and Preumont, 2012; Karásek, 2014) and modified our code using their simple hummingbird model simulator with permission for our control simulation. In this instance, using a controller was not necessary as our primary focus was to analyze the inherent dynamic stability of the PAV model, without the influence of external control. So this is an additional analysis to check whether the controller performs well. As previously stated, the code from (Karásek and Preumont, 2012; Karásek, 2014) was taken as reference and controller was designed and utilized here using Matlab/Simulink^®^for insect flight analysis. Some notable features of their controller model are: 1. Decoupling the system into three subsystems (longitudinal, vertical, lateral + yaw) 2. The controller has two main loops: the inner one for attitude stabilization and the outer one for controlling velocities 3. Discrete type of design for cycle average forces and moments 4. Velocities controlled by PI controllers that need to be appropriately tuned. Figure 8 depicts the flowchart diagram of their controller scheme.

It should be noted that the non-dimensional control derivative matrix is a critical component of this controller because it is responsible for converting or transforming the forces into the required wing motion. These control derivatives were obtained by us along with the stability derivatives using the same method described in the previous section and fed into the code. The sweep amplitude $\emptyset_{m}$ and offset $\emptyset_{0}$ are two essential control parameters in the code, and their value affects the force and moments generated inside. The primary change affecting the analysis is the kinematic behavior of the insect flight, such as stroke amplitude and flapping frequency, which are low and high, respectively, and must be adjusted as part of the controller. In real-world robot design, the number of parameters required to control flight must be kept to a minimum, and additional constraints reduce the effect of parameter changes on control force/moment (Karásek and Preumont, 2012). According to reference (Deng et al., 2006; Karásek and Preumont, 2012), Since we have symmetric wing kinematics, the transformation of control parameters $S_{1},S_{2},S_{3},\ldots$ into kinematic ones, takes place as shown in Eq. 30 below:

$${\left[S_{1},S_{2},S_{3},\ldots \right]}^{T}=J^{-1}{\left[\begin{array}{cccc} X & L & M & N \end{array}\right]}^{T}$$ (30)

Where J is the control derivative matrix (CDM). Furthermore, the control design is based on the linearized model, whereas the original system is nonlinear. This necessitates testing the control performance of each combination in nonlinear simulation (Karásek and Preumont, 2012).

The feasibility of each choice’s wing control mechanism design will also constrain the final selection of control parameters in the real robot. The controller performance results from this control simulator modified for insect flight are sufficient for preliminary assessment before a detailed high-fidelity CFD analysis can be implemented. These findings and the excellent analytical calculations for dynamic stability of the linearized model presented in this paper aided in the initial design considerations.

## 4 Results and discussion

### 4.1 System matrices and eigenvalues

Referring back to the dynamic stability analysis performed on the model as depicted in Sections 2, 3, the non-dimensionalized stability derivatives were then obtained, resulting in the construction of both the longitudinal and lateral system matrices as shown in Eqs 31, 32. A small 99-line Matlab^®^code was written to obtain the system matrices and the pole-zero plot from the data, observe the mode eigenvalue locations on the s-plane, and comment on stability.

$${A_{Long}}^{+}=\left[\begin{array}{cccc} -0.0019 & 0.0008 & -0.0013 & -0.0015\\ 0.0021 & -0.0053 & -0.0004 & 0\\ 0.0637 & -0.0125 & -0.0132 & 0\\ 0 & 0 & 1 & 0 \end{array}\right]$$ (31)

$${A_{Lat}}^{+}=\left[\begin{array}{cccc} -0.0029 & -0.0003 & 0.0010 & 0.0015\\ 0.0144 & -0.0431 & 0.0037 & 0\\ 0.0088 & -0.0082 & -0.0434 & 0\\ 0 & 1 & 0 & 0 \end{array}\right]$$ (32)

The system matrices are further solved to obtain the eigenvalues and to identify the longitudinal and lateral dynamic modes. This aids in comprehending the model’s dynamic stability in the hovering state. Figure 10 depicts the pole-zero plot (s-plane) representing all the eigenvalues and dynamic modes. Figure 11 depicts the velocity magnitude of flapping PAV wings at high frequency with r/R = 0.5 (50% span) at different instants of one complete flapping cycle. The body motion is neglected because of the hovering. At hover, the increase in non-dimensional vertical force ( $-{F_{Z}}^{+}$ ) during the downstroke also results in a lift coefficient that is slightly higher in magnitude during the downstroke than during the upstroke, which contributes significantly to cycle averaging components and thus supports the weight.

**FIGURE 9:**
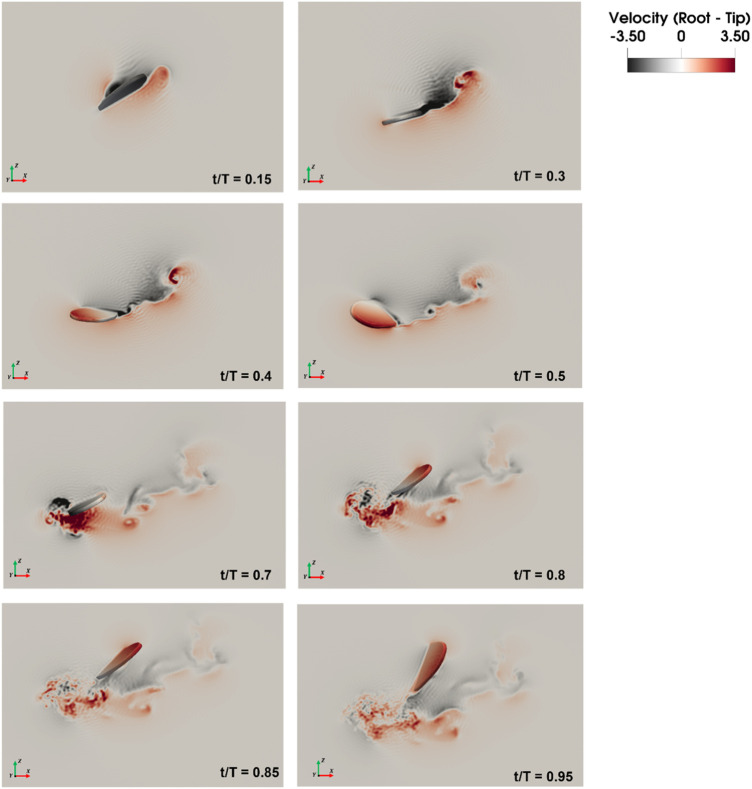
Velocity magnitude contours of the mosquito-inspired PAV wings flapping at high frequency (r/R = 0.5).

The numerical results in Figure 9 of the insect-inspired Pico Aerial Vehicle (PAV) flapping at high frequencies reveal promising outcomes. The heightened wingbeat frequencies, reminiscent of mosquito flight characteristics, contribute to unique aerodynamic effects. The aerodynamic analysis indicates the generation of trailing edge vortices through wake capture, reduced reliance on leading vortices, and the mitigation of rotational drag. In terms of energetics, the PAV’s performance during high-frequency flapping is noteworthy. The kinematic and quasi-steady aerodynamic modeling, considering translational, rotational, and wake capture forces, demonstrates efficient energy utilization. The adaptation of control strategies from bird flapping wings to insect wing kinematics proves effective, influencing the wing forces and providing a solid foundation for flight controller design.

Dynamic stability analysis further underscores the favorable characteristics of the mosquito-inspired PAV during high-frequency flapping. Despite its small scale, the PAV exhibits a robust controller response, demonstrating excellent maneuverability. The modified model, incorporating rigid body dynamics and non-averaged aerodynamics, reveals weak stability without a controller or insufficient power density. However, the controller promptly stabilizes the PAV, ensuring its reliable performance in terms of attitude control and maneuverability. These inference results not only contribute to a deeper understanding of the aerodynamics and energetics of insect-inspired flapping-wing flight but also underscore the potential of such high-frequency flapping in enhancing the capabilities of Pico Aerial Vehicles for various applications.

**FIGURE 10:**
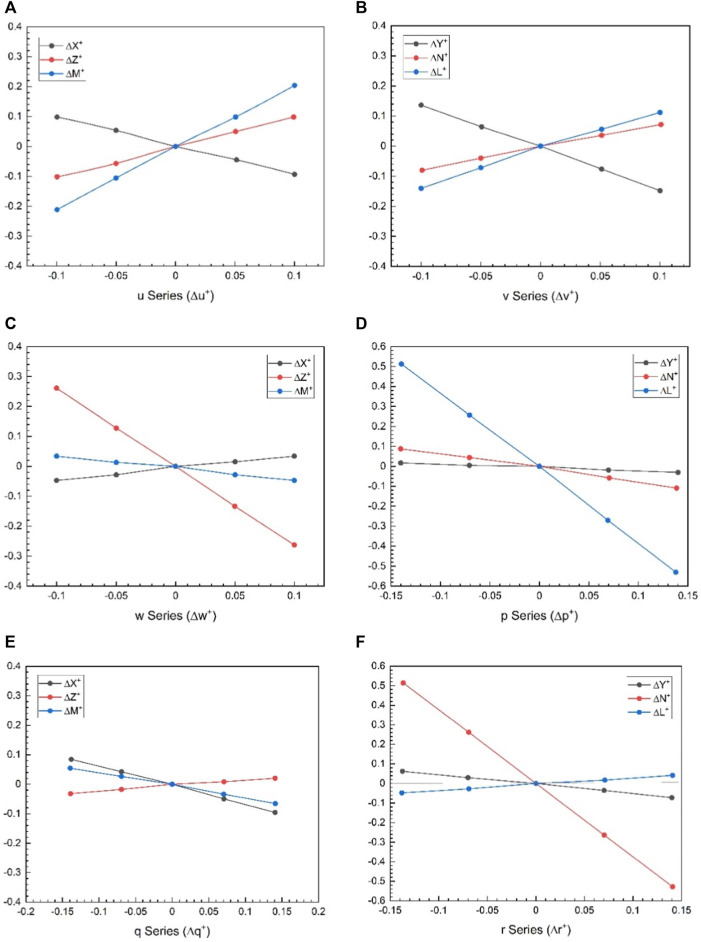
Time variations of the aerodynamic forces and moment of the wings in a flapping period including equilibrium flight; **(A)** u -series - data (for values of ${X_{u}}^{+}$ , ${Z_{u}}^{+}$ and ${M_{u}}^{+}$ ); **(B)** v -series - data (for values of ${Y_{v}}^{+}$ , ${N_{v}}^{+}$ and ${L_{v}}^{+}$ ); **(C)** w -series - data (for values of ${X_{w}}^{+}$ , ${Z_{w}}^{+}$ and ${M_{w}}^{+}$ ); **(D)** p -series - data (for values of ${Y_{p}}^{+}$ , ${N_{p}}^{+}$ and ${L_{p}}^{+}$ ); **(E)** q -series - data (for values of ${X_{q}}^{+}$ , ${Z_{q}}^{+}$ and ${M_{q}}^{+}$ ); **(F)** r -series - data (for values of ${Y_{r}}^{+}$ , ${N_{r}}^{+}$ and ${L_{r}}^{+}$ ).

At the equilibrium flight conditions, the aerodynamic forces and moments acting on the wings are computed for each of the parameters ( u,v, and w ) and ( p,q , and r ) with variations independently deviating from their equilibrium values. The corresponding values for each derivatives are then determined. In Figures 10A–F, the data for the all these series are graphically represented. This observation suggests that, for small disturbance motions, the linearization of aerodynamic forces and moments is well-justified.

**FIGURE 11:**
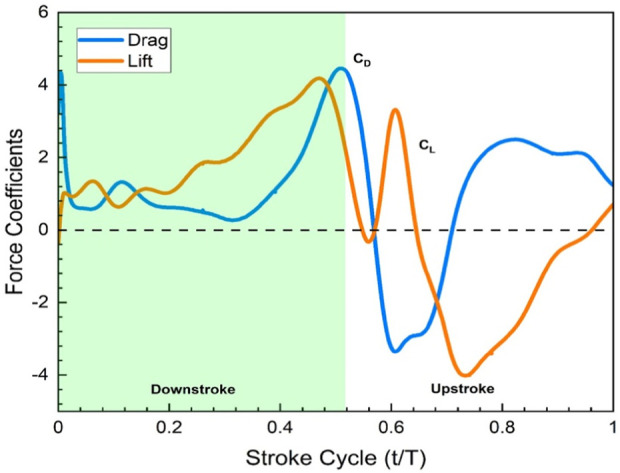
Force Coefficients , (wing contribution) in a hovering state.

Figure 11 reveals that drag and lift show good convergence and typically represent the aerodynamic behavior of flapping wings. This is due to the same fact that the leading edge vortex is developed precisely at the beginning of the downstroke and intensifies to its maximum during the middle of the downstroke (t/T = 0.45), because of which there are peaks in the lift force. At the rotation point (t/T = 0.55) between the two strokes, the step rotation in the wings results in large peaks in the aerodynamic forces.

During the downstroke after pronation, the wing forms a trailing edge vortex (TEV), creating a negative pressure region that boosts lift. Subsequent strokes, including forward stroke with supination and upstroke after supination, involve both TEV and tip vortices, contributing to lift peaks. Positive pressure near the trailing edge of the ventral surface occurs separately, representing distinct mechanisms. Interestingly, the leading-edge vortex (LEV) negatively affects lift at the second peak, receding before supination. In contrast, TEV and tip vortices play key roles in the second lift peak. Throughout the downstroke, force and lift coefficient remain significant, with a dip at the second half of the upstroke.

**FIGURE 12:**
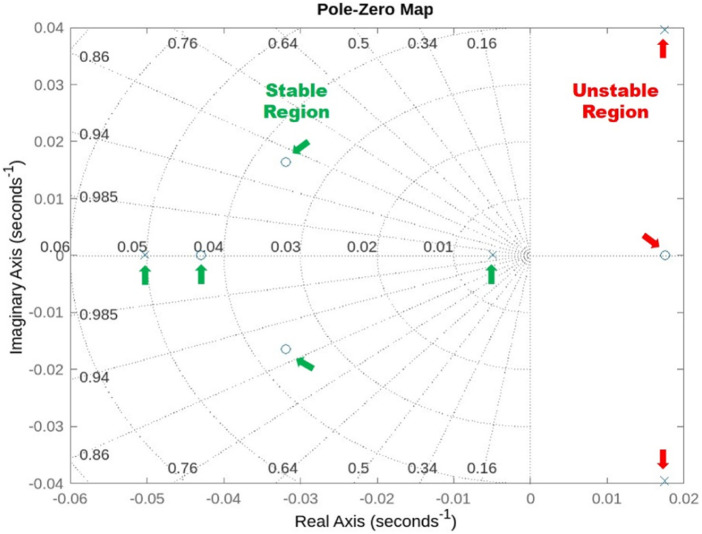
Representation of longitudinal and lateral eigenvalues with information about stable and unstable dynamic modes in 2D s-plane.

At the hovering state (0 m/s), one unstable oscillatory dynamic motion mode with complex eigenvalues on the right half of the s-plane and two stable real modes with negative real eigenvalues on the left stable half of the s–plane have been observed. As a result, the mosquito-inspired PAV is weakly stable in longitudinal motion. Despite having stable oscillatory and subsidence modes, the model is also weakly stable in the lateral mode due to one of the positive real eigenvalues. This analysis demonstrates that the authors accurately captured the kinematics of real mosquitos for their PAV. Because of the weak stability in both the longitudinal and lateral cases, it is recommended that the PAV be built with an effective controller onboard or, to a minimum, an efficient actuation system capable of generating enough power density to lift off, hover, and counteract this effect. Figure 12 represents the stable and unstable eigenvalues plotted on an s-plane (pz map), clearly indicating instability in hover and a need for a controller. Tables 4, 5 show the eigenvalues obtained from this analysis and their comparability with eigenvalues from other studies for both longitudinal and lateral cases.

**TABLE 4:** Longitudinal stability modes (Eigenvalues from system matrices) in hovering.

Model^a^[reference]	Longitudinal stability (eigenvalues) in hover			
	$\lambda_{1}$	$\lambda_{2}$	$\lambda_{3}$	$\lambda_{4}$
Drone fly (Zhang and Sun, 2010; Zhu et al., 2020)	0.0469 + 0.0967i	0.0469–0.0967i	−0.1196	−0.0139
Bumblebee (Xiong and Sun, 2008; Xu and Sun, 2013; Zhu et al., 2020)	0.0450 + 0.1290i	0.0450–0.1290i	−0.1970	−0.0120
Male Mosquito (Liu and Sun, 2019)	0.0178 + 0.0355i	0.0178–0.0355i	−0.0435	−0.0082
Female Mosquito (Liu and Sun, 2019)	0.0227 + 0.0445i	0.0227–0.0445i	−0.0543	−0.0075
RoboMos PAV Model	0.0174 + 0.0396i	0.0174–0.0396i	−0.0503	−0.0049

^a^ Dronefly ( f = 178 Hz), Bumblebee ( f = 155 Hz), and our model all have almost the same size but differ in kinematics from our model. Our wing kinematics are identical to those of MM ( f = 777 Hz) and FM ( f = 572 Hz), but the size differs from our model.

**TABLE 5:** Lateral stability modes (Eigen Values from system matrices) in hovering.

Model^a^[reference]	Lateral stability (eigenvalues) in hover			
	$\lambda_{1}$	$\lambda_{2}$	$\lambda_{3}$	$\lambda_{4}$
Drone fly (Zhang and Sun, 2010; Zhu et al., 2020)	0.0478	−0.0779 + 0.0504i	−0.0779–0.0504i	−0.5118
Bumblebee (Xiong and Sun, 2008; Xu and Sun, 2013; Zhu et al., 2020)	0.0940	−0.1180 + 0.0720i	−0.1180–0.0720i	−0.6860
Male Mosquito (Liu and Sun, 2019)	0.0203	−0.0250 + 0.0156i	−0.0250–0.0156i	−0.0390
Female Mosquito (Liu and Sun, 2019)	0.0353	−0.0380 + 0.0353i	−0.0380–0.0353i	−0.0216
RoboMos PAV Model	0.0176	−0.0320 + 0.0164i	−0.0320–0.0164i	−0.0430

^a^ Dronefly ( f = 178 Hz), Bumblebee ( f = 155 Hz), and our model all have almost the same size but differ in kinematics from our model. Our wing kinematics are identical to those of MM ( f = 777 Hz) and FM ( f = 572 Hz), but the size differs from our model.

The results of modes in Tables 4, 5 also show greater consistency in terms of both longitudinal and lateral dynamic modes with previous studies and mode eigenvalues that are conventionally consistent with those of other real insects and models, even though they all have different flapping frequencies and amplitudes. In each case, stability derivatives govern these mode configurations.

It is interesting to note an important observation, which is not investigated here but shall be implemented experimentally for this model in the future. According to the reference (Liu and Sun, 2019), spreading the legs during the flight affects the moment of inertia but has a minor effect on the mosquito’s dynamic stability. Because the mosquito-inspired PAV developed in the laboratory has a detachable leg system, this observation will assist in carefully analyzing the flight in terms of stability during real-time flight tests and experiments in the wind tunnel.

### 4.2 Controller performance

**FIGURE 13:**
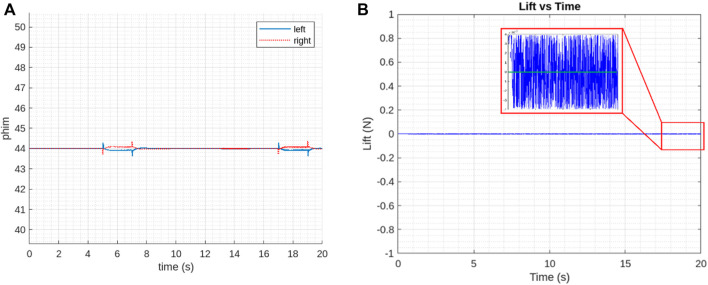
Controller performance **(A)** controlling factor, sweep amplitude $\phi_{m}$ **(B)** Vertical Force measurement for generated and desired response.

The modified version of the model simulator with help from reference (Karásek and Preumont, 2012) used here in this study resulted in the evaluation of controller performance with step inputs, considering the state vectors $V\to f\left(u,v,w\right)$ and r . Mean stroke angle (sweep) amplitude $\phi_{m}$ is the controlling parameter. However, as shown in Figure 13A, the sweep amplitude is well within the control limits ∼ 44°. With each step command applied to the system, decoupling is observed between longitudinal and lateral dynamics, suggesting similarity as per the linear calculations.

The generated vertical force is well controlled by the controller. The drift in its desired value at the end as shown in Figure 13B, might be due to the process of determination of control derivatives for CDM. This is in accordance with the trajectory that the PAV followed during hovering. However, the mean value of the parameters still closely follows the linear results with PAV kinematics.

The controller’s performance in the lateral direction behaves in the same way. Since the linearized dynamic analysis already gave good results, this preliminary controller performance assessment is enough to exhibit the need for a suitable controller.

Although the control parameters should be zero in hovering flight according to the linearized model, it is observed that sweep amplitude $\phi_{m}$ is slightly drifted compared to the desired value at certain points of time when all commands are zero. The explanation is the same as in reference (Karásek and Preumont, 2012) that the flapping motion induces a body oscillation that changes the wing’s velocity and the angle of attack. This results in a slight increase of the cycle-averaged lift force (desired), as shown in 9B, which is then compensated by decreasing the sweep amplitude (brought back to zero).

**FIGURE 14:**
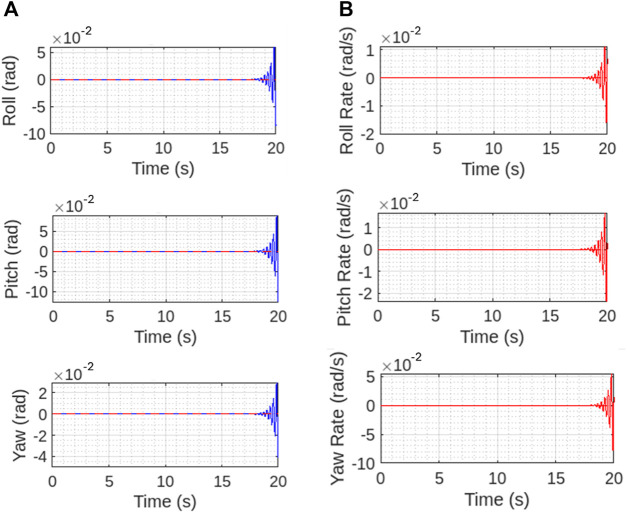
Controller performance for **(A)** maneuvering angles and **(B)** angular rates. The controller performance is well withing the reference till at the mid hovering state and controls the vehicle.

Figures 14A, B show the controller performance in case of attitude and attitude rates. The interpretation of graphs shows slight differences between reference and generated results for controller performance in all angular rates and maneuvering that happens as vehicle reaches the hovering state and involves a thorough analysis of the magnitude, patterns, stability, and response time of these differences. This analysis helps in assessing the controller’s performance and guiding improvements to ensure that the system operates as close to the desired values as possible.

**FIGURE 15:**
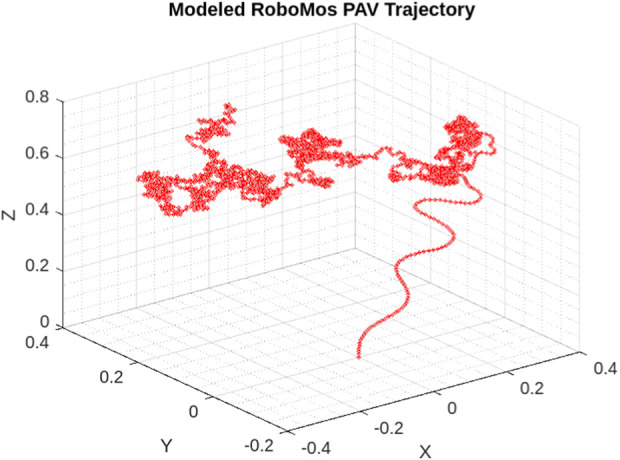
**3D**Representation of RoboMos model trajectory from take off to hovering. Controller performance is depicted during the flight.

**FIGURE 16:**
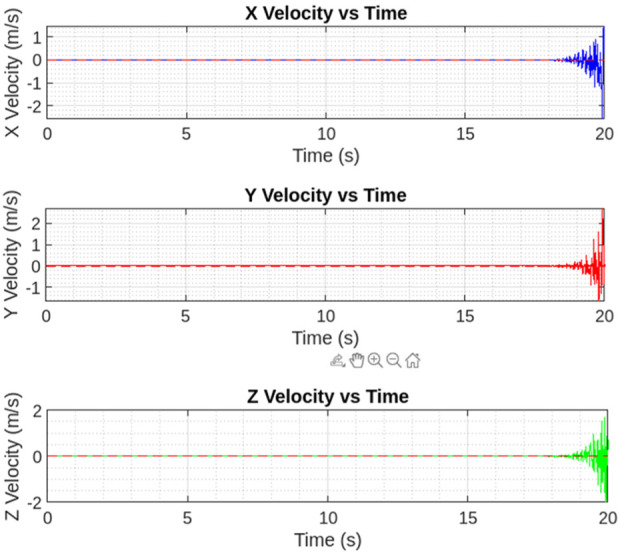
Three-dimensional velocity (state) plots. Again, the controller performance is well in accordance to the reference signal (red dashed).

Figure 15 shows the desired trajectory that PAV follows during the take off and then hovering. As depicted the controller is well responsive during both the phases and keeps the vehicle balanced in hovering state implemented. Figure 16 shows the desired velocities in three directions with controller after some fine tuning. This clearly signifies that this insect inspired PAV can create complex wing trajectories using power and steering attached to the lifting surface and its actuation system. It is obvious that flight control such as stable hovering and trajectory tracking of actual fabricated PAV is a challenging task.

## 5 Conclusion

The primary objective of this research was to conduct an initial assessment of the modeling, stability, and control aspects of RoboMos, a pico aerial vehicle prototype inspired by mosquitoes. The aim was to closely mimic the natural morphology and flight characteristics of actual mosquitoes. This analysis yielded valuable preliminary insights, encompassing mathematical modeling and control analysis. It precedes and helped with more detailed 3D computational analysis and the completion of the physical prototype for real-time flight testing. The key findings from this study are as follows:

• The pico aerial model closely resembles the natural structure and, to a large extent, the aerodynamics of a real Mosquito. In terms of longitudinal motion, it exhibits one unstable oscillatory mode and two stable subsidence modes. There is an unstable divergence mode, a stable oscillatory mode, and a stable subsidence mode for lateral motion.
• Despite several peculiarities in the aerodynamic mechanisms of mosquitoes, the major aerodynamic properties remain consistent.
• Due to the weak stability in both longitudinal and lateral motions, it is recommended that the PAV be equipped with an effective onboard controller or, at a minimum, an efficient actuation system capable of providing sufficient power density for takeoff, hovering, and counteracting instability.
• This analysis affirms that we accurately captured the kinematics of real mosquitoes in their PAV model. We hope that the fabricated RoboMos PAV will exhibit stable flight during real-time flight tests.
• The controller performance was thoroughly evaluated and it was found that vehicle attitude is controlled well by the controller during each phase of PAVs flapping flight.

## Data Availability

The datasets presented in this article are not readily available because data can be acquired through an appropriate request. Requests to access the datasets should be directed to BS, balbir.s@manipal.edu.
